# N-Glycomic Profiling of Microsatellite Unstable Colorectal Cancer

**DOI:** 10.3390/cancers15143571

**Published:** 2023-07-11

**Authors:** Iiris Ukkola, Pirjo Nummela, Annamari Heiskanen, Matilda Holm, Sadia Zafar, Mia Kero, Caj Haglund, Tero Satomaa, Soili Kytölä, Ari Ristimäki

**Affiliations:** 1 HUSLAB, Department of Pathology, HUS Diagnostic Center, Helsinki University Hospital and University of Helsinki, 00029 Helsinki, Finland; 2 Applied Tumor Genomics Research Program, Research Programs Unit, Helsinki University Hospital and University of Helsinki, 00014 Helsinki, Finland; 3 Glykos Finland Co., Ltd., 00790 Helsinki, Finland; 4 Translational Cancer Medicine Research Program, Faculty of Medicine, University of Helsinki, 00014 Helsinki, Finland; 5 Department of Surgery, Helsinki University Hospital and University of Helsinki, 00029 Helsinki, Finland; 6 HUSLAB, Department of Genetics, HUS Diagnostic Center, Helsinki University Hospital and University of Helsinki, 00029 Helsinki, Finland

**Keywords:** BRAF^V600E^, colorectal cancer, microsatellite instability, mass spectrometry, N-glycosylation

## Abstract

**Simple Summary:**

All human cells possess a complex glycan coating, and alterations in cell surface glycans play a role in cancer progression and immune suppression. Colorectal cancer is a heterogenous disease that can be classified into several molecular subtypes, with major variances in prognosis and therapy responses. Two important molecular markers in this regard are microsatellite instability and *BRAF* gene mutation, which have been largely ignored in previous glycomics studies. We analyzed the N-glycan profiles of local and advanced colorectal cancers consisting of different molecular subtypes to identify possible explanations for their differing behaviors. Our results show that the studied molecular subgroups of colorectal cancer exhibit characteristic glycan profiles, which may explain their tumorigenic properties.

**Abstract:**

Aberrant glycosylation affects cancer progression and immune evasion. Approximately 15% of colorectal cancers (CRCs) demonstrate microsatellite instability (MSI) and display major differences in outcomes and therapeutic responses, as compared to corresponding microsatellite stable (MSS) tumors. We compared the N-glycan profiles of stage II and IV MSI CRC tumors, further subdivided into BRAF^V600E^ wild-type and mutated subgroups (*n* = 10 in each subgroup), with each other and with those of paired non-neoplastic mucosal samples using mass spectrometry. Further, the N-glycans of BRAF^V600E^ wild-type stage II MSI tumors were compared to corresponding MSS tumors (*n* = 9). Multiple differences in N-glycan profiles were identified between the MSI CRCs and control tissues, as well as between the stage II MSI and MSS samples. The MSI CRC tumors showed a lower relative abundance of high-mannose N-glycans than did the control tissues or the MSS CRCs. Among MSI CRC subgroups, acidic N-glycans showed tumor stage and BRAF mutation status-dependent variation. Specifically, the large, sulfated/phosphorylated, and putative terminal N-acetylhexosamine-containing acidic N-glycans differed between the MSI CRC subgroups, showing opposite changes in stages II and IV, when comparing BRAF mutated and wild-type tumors. Our results show that molecular subgroups of CRC exhibit characteristic glycan profiles that may explain certain carcinogenic properties of MSI tumors.

## 1. Introduction

Colorectal cancer (CRC) is a heterogenous disease that can be stratified by genomic and gene expression profiles into several distinct molecular subtypes, with major differences in prognosis and therapeutic response. Approximately 15% of CRCs arise from a microsatellite instability (MSI) pathway caused by a deficient mismatch repair (dMMR) system, leading to hypermutation and increased cancer susceptibility. The MMR system can be compromised by epigenetic changes, usually by acquired *MLH1* promoter hypermethylation, or by genetic inactivation of *MLH1*, *MSH2*, *MSH6*, or *PMS2* genes characteristic of Lynch syndrome (LS) [1,2]. Among all CRC tumors, stage II–III MSI CRCs have a better prognosis than corresponding microsatellite stable (MSS) tumors, whereas stage IV MSI CRCs exhibit a worse prognosis than corresponding MSS CRCs [1,2]. Further, *BRAF*^V600E^ mutation has been associated with aggressive behavior in MSS tumors, but not in MSI tumors, suggesting that the MSI phenotype may override the negative prognostic potential of *BRAF*^V600E^ mutation [3,4,5,6]. Immuno-oncological treatments with immune checkpoint inhibitors have shown excellent responses in advanced dMMR/MSI CRC patients [7,8] but only limited responses when treating MSS CRC patients [9,10]. Despite the clinical success, nearly half of metastatic dMMR/MSI CRCs fail to respond to immunotherapy [11,12]. The molecular mechanisms behind these differing responses are unclear.

All human cells have a complex glycan coating (the glycocalyx), which is involved in many essential cellular functions, such as cell signaling, adhesion, differentiation, and proliferation [13,14]. Altered glycosylation, most often increased sialylation, fucosylation, and branching of N-linked glycans, has been observed in many types of cancer cells [13,14]. Aberrantly expressed glycans can modulate essential events of cancer development and progression, including angiogenesis, invasion, and metastasis [13,14,15]. Further, the surface glycans of cancer cells play a role in the evasion of the immune response [16]. Glycan alterations may thus provide valuable novel molecular targets for cancer diagnostics and treatment.

N-glycan profiles of CRC tissues have previously been compared primarily with those of non-neoplastic colon tissues, and different stages of CRC have been compared, without considering the MSI status. Increased glycosylation features in CRC include pauci-mannosidic, β1,6-branched, sulfated and sialylated N-glycans, especially α2,6-sialylated glycans, as well as glycans containing sialylated Lewis type epitopes [17,18]. In turn, the decreased features include structures with a bisecting N-acetylglucosamine [17,18]. In our previous study, we also demonstrated differences in the levels of sialylated and sulfated glycans between stage II and III right-sided CRC samples, with stage III tumors showing predominantly sulfated and stage II exhibiting mainly sialylated N-glycans [19]. Also, a study by Kaprio et al. showed pauci-mannose and small high-mannose N-glycan structures, as well as sialylated structures, to be relatively more abundant in rectal carcinomas than in adenomas [20].

The aim of this study was to analyze the N-glycan profiles of MSI CRC tumors to identify possible reasons for their differing behaviors. We studied the neutral and acidic N-linked glycan profiles of MSI CRC samples (n = 40) and pools of paired non-neoplastic colon controls (n = 4) using MALDI-TOF mass spectrometry. The MSI CRC samples were further divided into subgroups according to the stage (II or IV) and BRAF^V600E^ mutation status (wt or mut) (n = 10 in each group). In addition, the N-glycan profiles of stage II BRAFwt MSI tumors were compared to those of stage II BRAF^V600E^ wt MSS tumors (n = 9).

## 2. Materials and Methods

### 2.1. Tissue Samples

Representative tissue samples from 40 MSI CRC patients were selected for the analysis. Of these patients, 38 had undergone surgical resection at Helsinki University Hospital (HUH) between 2018 and 2019, and the samples had been routinely screened for the MMR proteins MLH1, MSH2, MSH6, and PMS2 using immunohistochemistry (IHC). The selected samples showed loss of MLH1 expression (and concomitant loss of PMS2) and had also been routinely screened by BRAF^V600E^ IHC in a real-life diagnostic setting. In addition, two MSI stage IV CRC samples from patients receiving surgery at HUH between 2014 and 2015 were included in the study. These samples were also determined to be dMLH1 by IHC, and the *BRAF*^V600E^ status had been analyzed by next generation sequencing (NGS). The primary selection of MSI (dMLH1) CRC samples was performed based on tumor stage (II or IV) and BRAF^V600E^ mutation status (mut or wt). Secondary selection was conducted based on patient age, sex, and tumor location (right or left side) to achieve similar study groups. For each of the MSI CRC subgroups (BRAFwt stage II, BRAFwt stage IV, BRAFmut stage II, and BRAFmut stage IV), 10 samples were selected. In addition to the BRAF^V600E^ IHC, the *BRAF* mutation status was confirmed by NGS in stage IV CRC samples and by droplet digital polymerase chain reaction (ddPCR) in stage II samples. Further, a pool of paired non-neoplastic colon samples from each MSI CRC patient set (four pools, n = 10 in each pool) was included in the glycomic profiling to serve as control tissues. For MSS reference sample set, we used our previously analyzed stage II MSS CRC samples [19]. From this published cohort, we selected only the stage II pMMR/MSS and BRAFwt cases (n = 9) by using MMR and BRAF^V600E^ IHC. These MSS stage II CRC patients had been operated on at HUH between 2001 and 2003.

### 2.2. BRAF Mutation Analysis

*BRAF*^V600E^ mutation status was confirmed by NGS in stage IV MSI CRC samples and by ddPCR in stage II MSI CRC samples. NGS analysis was performed using an in-house cancer panel containing the *BRAF* exons 11–15 (in addition to the coding regions of *PIK3CA*, *EGFR*, *KIT*, *KRAS*, *MET*, *NRAS*, and *PDGFRA*), performed as previously described [21].

For the ddPCR analysis, targeted wild-type and *BRAF*^V600E^ mutation probes were designed and prevalidated by Bio-Rad (www.biorad.com), and 2 μL (100 ng) of the extracted DNA from formalin-fixed paraffin-embedded (FFPE) tissue samples was used for each duplicate reaction. Droplet generation and reading were performed according to the manufacturer’s protocol using QX200 Droplet Generator and QX200 Droplet Reader (Bio-Rad, Hercules, CA, USA), respectively. The droplet generator first partitioned the samples for PCR amplification (22 μL into 20,000 droplets), and the droplets from each sample were then analyzed individually on the droplet reader. The data were processed using the QuantaSoft Analysis Pro Software (v.1.0; Bio-Rad).

### 2.3. N-Glycan Isolation

Representative areas of the carcinoma tissue, or areas containing the highest percentages of epithelial cells in the paired non-neoplastic colon tissues, were marked on HE slides, and macrodissection was used to cut 10 µm thick flakes from the FFPE tissue blocks with a Leica SM2000R microtome (Leica Microsystems GmbH, Wetzlar, Germany). After the last flake was obtained, a new HE slide was stained to verify the representativeness of the flakes. The tissue flakes were deparaffinized with xylene and rehydrated with a descending ethanol series, according to standard procedures, and N-linked glycans were liberated by N-glycosidase F (PNGase F) digestion (Glyko; ProZyme Inc., Hayward, CA, USA) overnight at 37 °C. N-glycan purification was then conducted, as previously described [20], using the 96-well format. Briefly, the extracted glycans were passed through C_18_ silica in water and then absorbed into graphitized carbon material. The carbon wells were washed with water, and neutral N-linked glycans were eluted using 25% acetonitrile and acidic N-linked glycans with 0.05% trifluoroacetic acid in 25% acetonitrile in water. The acidic N-glycans were further purified with hydrophilic interaction solid-phase extraction. Both glycan fractions were then additionally passed through strong cation-exchange resin and C_18_ silica resin in water.

### 2.4. Mass Spectrometry

Matrix-assisted laser desorption/ionization time-of-flight (MALDI-TOF) mass spectrometry (MS) was performed using a Bruker Ultraflex III TOF/TOF instrument (Bruker Daltonics Inc., Bremen, Germany), as previously described [20]. Neutral N-linked glycans were detected in positive ion reflector mode as (M+Na)^+^ ions, and acidic N-linked glycans were identified in negative ion reflector mode as (M-H)^−^ ions. Representative unprocessed MALDI TOF mass spectra of neutral and acidic N-linked glycans are shown in Supplementary Figure S1. The relative molar abundances of both neutral and acidic N-glycan components were assigned based on their relative signal intensities in the mass spectra when analyzed separately as neutral and acidic glycan fractions. The unprocessed mass spectrometric data were first transformed into the present glycan profiles by removing the effects of isotopic pattern overlapping, alkali-metal adduct signals, water elimination products from reducing oligosaccharides, and other interfering mass spectrometric signals not arising from the original glycans in the sample, as previously described [22,23]. The resultant glycan signals in the glycan profiles were then normalized to 100% to allow for the relative quantitative comparison between the samples. Normalized values were further assigned to structural/biosynthetic glycan classes based on their proposed monosaccharide composition, as previously described [22,23]. The validation of proposed monosaccharide compositions identified by this method in colon and lung carcinoma specimens has been presented in the previous study by Satomaa et al. [22]. Further, proposed monosaccharide compositions and identified N-glycan features of CRC tumor specimens have also been validated in a detailed study by Balog et al. [17]. All the glycan signal intensities and relative abundances of both glycans and glycan classes identified in this study are listed in Supplementary Tables S1 and S2. The mass spectrometry proteomics data from the previously analyzed stage II BRAF^V600E^ wt MSS CRC samples have been deposited in the ProteomeXchange Consortium via the PRIDE [24,25] partner repository, with the dataset identifier PXD018673 (samples AH25-31-21-1/2, AH25-31-8-1/2, AH25-31-12-1/2, AH25-31-20-1/2, AH25-31-4-1/2, AH25-31-13-1/2, AH25-31-9-1/2, AH25-31-5-1/2, and AH25-31-37-1/2).

### 2.5. Analysis of N-Linked Glycan Profiles

In seven different study group settings, statistical analysis of the N-linked glycan data was performed between non-neoplastic colon samples (four pools of paired control samples), subgroups of MSI CRC samples, according to the tumor stage and BRAF mutation status, and stage II BRAF^V600E^ wt MSI CRCs were compared to stage II BRAF^V600E^ wt MSS tumors (Table 1). Both the relative abundances of proposed monosaccharide compositions and glycan classes were analyzed separately within these study groups. For statistical analyses, the nonparametric Kruskal–Wallis test was first used to verify the equality of the mean ranks of the groups, and the Mann–Whitney U test, along with the Benjamini–Hochberg false discovery rate (FDR) correction method [26], was then used for pairwise comparisons of the groups. RStudio (version 2022.07.2-576; Posit Software, Boston, MA, USA) was used for statistical analyses, and *p*-value < 0.05 was considered statistically significant. The results are shown as mean relative abundance ± standard error of mean (SEM) for the separately calculated neutral and acidic N-glycan compositions and structural glycan classes.

### 2.6. Immunohistochemistry

For IHC analyses, 4 μm sections cut from the FFPE tissue blocks were used. The MMR and BRAF^V600E^ IHC were performed, as previously described [27,28].

## 3. Results

### 3.1. Clinicopathological Characteristics of the CRC Cases

Our study consisted of 40 MSI CRC samples, which were subdivided into four different subgroups (*n* = 10 in each group) according to tumor stage (II or IV) and BRAF^V600E^ mutation status (wt or mut) (Table 1). Of these patients, the median age was 73 (range 51–89), 70% were females, and the tumors were mainly localized to the right colon (80–100%), which are typical characteristics of MSI CRC patients (Table 2). MSI stage II tumors were mainly pT3 (70–80%) and low-grade (60%). MSI stage IV BRAFwt CRCs were more often high-grade (60% vs. 30%) and pT4 (60% vs. 30%), more often displaying peritoneal metastasis (M1c, 40% vs. 10%) when compared to MSI stage IV BRAFmut CRCs (Table 2). Stage IV MSI CRCs presented mainly synchronous metastases (16/20, 80%), and four cases were diagnosed with metachronous metastases at less than 6 months (2–5 months) follow-up. In the MSS stage II BRAFwt subgroup (*n* = 9), a slight predominance of male sex (56%) and left-sided localization (56%) was observed, and the median age of the patients was 59 (range 52–77). Similar to the results for MSI stage II CRCs, the MSS tumors were mainly pT3 (89%) and low-grade (78%) (Table 2).

### 3.2. N-Linked Glycan Profiles

#### 3.2.1. N-Glycan Profiles of MSI CRC Samples and Paired Non-Neoplastic Control Samples

**Figure 1:**
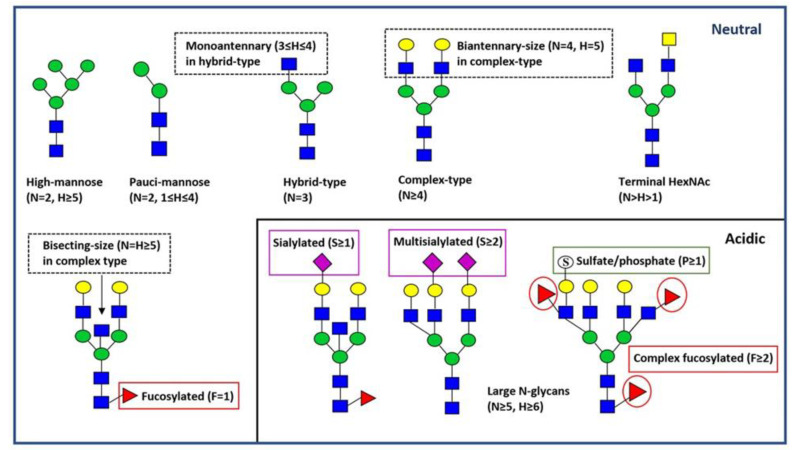
Major types and features of N-glycans. Representative N-glycan structures are depicted by green circles (D-mannose), blue squares (N-acetyl-D-glucosamine), yellow squares (N-acetyl-D-galactosamine), red triangles (L-fucose), yellow circles (D-galactose), purple diamonds (N-acetylneuraminic acid, sialic acid), and open circles with an “S” (sulfate or phosphate ester). The major N-glycan structural classes are shown below the schematic structures. Additional N-glycan structural features are indicated in boxes. H = hexose; N = N-acetylhexosamine = HexNAc; F = deoxyhexose (fucose), S = sialic acid; P = acid ester (sulfate/phosphate).

The major glycan types and structural features reported in this study are shown in Figure 1. Most of the differences between paired non-neoplastic colon samples (four pools) and MSI CRC samples (*n* = 40) were detected in the neutral N-glycan profiles. Relative abundances of pauci-mannose, biantennary-size complex-type, monoantennary hybrid-type, fucosylated pauci-mannose (especially H2N2F1), and fucosylated hybrid-type glycans were significantly higher in the MSI tumor samples than in the controls (Table 3). Respective comparisons of proposed monosaccharide compositions are shown in Supplementary Table S3. In contrast, the relative abundances of five N-acetylhexosamines (HexNAc = N) containing glycans (i.e., complex-type), high-mannose type glycans (e.g., compositions H6N2, H9N2, and H10N2), and a putative terminal HexNAc (N > H > 1) containing glycans were significantly lower in the MSI tumor samples than in the controls (Table 3 and Supplementary Table S3). In addition, the complex-type glycan structures with fucose and putative terminal HexNAc, e.g., H3N5F1 and H4N5F1, as well as the putative bisecting (N = H ≥ 5) N-acetylglucosamine structures H5N5F1 and H5N5F2, showed decreased abundances in MSI CRC as compared to the controls (Supplementary Table S3). In the acidic N-glycan profiles, only fucosylated complex-type glycans showed significant change, i.e., a decrease in MSI CRCs as compared to the controls (Table 3). Further, several multifucosylated and putatively sulfated N-glycan structures (e.g., H5N4F3P1, H4N5F3P1, H5N5F2P1, H5N5F3P1, and H6N6F3P1) were less abundant in the MSI tumor samples (Supplementary Table S3).

#### 3.2.2. N-Glycan Profiles between Stage II MSI and MSS Samples

The neutral N-glycan profiles of MSI BRAFwt stage II CRC samples (*n* = 10) were clearly different from those of our MSS BRAFwt stage II samples (*n* = 9). The MSI stage II CRC samples showed distinctively higher relative abundances of several neutral N-glycan classes, including complex-type glycans (4 HexNAc and 5 HexNAc), biantennary-size complex-type glycans, monoantennary hybrid-type glycans, and fucosylated glycans (particularly fucosylated pauci-mannose type glycans, e.g., H2N2F1), than did the MSS samples (Table 4). Further, the putative terminal HexNAc containing glycan H3N4F1 and fucosylated complex-type glycans H4N4F1, H5N4F1, H5N5F1, and H6N5F1 were more abundant in the MSI than in MSS samples (Supplementary Table S4). On the contrary, the MSI Stage II CRC samples showed lower abundances of oligomannose structures (N2 = 2 HexNAc, e.g., high-mannose type H5N2, H6N2, H7N2, and H8N2, and pauci-mannose type H2N2, H3N2, and H4N2), hybrid-type (3 HexNAc), and high-mannose type structures, as well as putative terminal HexNAc containing complex-type glycans, than did the MSS Stage II CRC (Table 4 and Supplementary Table S4).

Acidic N-glycan profiles of the MSI BRAFwt stage II CRC samples vs. the corresponding MSS cases showed a higher number of significantly different glycan classes than the neutral N-glycan profiles. Four HexNAc containing (N4) and biantennary-size complex-type N-glycans were relatively more abundant in the MSI stage II CRC samples than in the MSS stage II (Table 5), as well as the sialylated and multisialylated complex-type glycans S1H5N4F1, S1H5N4F2, S2H5N4, S2H5N4F1, S3H6N5, and S3H6N5F1 (Supplementary Table S5). Among the less abundant N-glycan classes in the MSI stage II as compared to MSS stage II samples were hybrid-type glycans (3 HexNAc) and large N-glycans (≥5 HexNAc), as well as fucosylated and complex fucosylated glycans (both features particularly noted in complex-type glycans, as well as complex fucosylation noted in the hybrid-type), putative terminal HexNAc structures (especially in the complex-type and bisecting size complex-type glycans), and acid ester-modified (sulfated/phosphorylated) hybrid-type N-glycans (Table 5).

#### 3.2.3. N-Glycan Profiles between BRAFwt and BRAFmut Stage II MSI Samples

When MSI stage II BRAFwt cases were compared to corresponding BRAFmut samples, only minor differences were observed in neutral N-glycan profiles, the fucosylated hybrid-type structures H2N3F1 and H3N3F1 being slightly increased in the BRAFmut samples (Supplementary Table S6). The acidic N-glycan profiles in turn displayed more differences, with the BRAFmut samples showing increased relative abundances of 4 HexNAc and biantennary-size complex type glycans, as well as sialylated complex-type glycans (especially S2H5N4) (Table 6 and Supplementary Table S7). Among the less abundant glycan classes in the BRAFmut stage II MSI samples were large N-glycans (≥5 HexNAc) and putative terminal HexNAc containing glycans, especially terminal HexNAc in complex-type and bisecting size complex-type glycans. (Table 6). Interestingly, no sulfated/phosphorylated N-glycans were identified in any of the BRAFmut stage II MSI CRC samples, and all detected acidic N-glycans were sialylated (Table 6).

#### 3.2.4. N-Glycan Profiles between Stage II and Stage IV MSI Samples

Only minor changes were observed in the neutral or acidic N-glycan profiles between all MSI stage II samples and MSI stage IV samples (*n* = 20 in both groups). In the neutral profiles, slight relative increases in hybrid-type glycans and fucosylated high-mannose type glycans, as well as the composition H3N4 (complex-type with putative terminal HexNAc), were detected in stage IV samples, whereas the composition H7N6F1 (fucosylated complex-type) showed a slight decrease in stage IV samples (Table 7 and Supplementary Table S6). When acidic N-glycan classes were compared, only biantennary-size complex-type glycans showed a relative decrease in stage IV samples (Table 6). Of the separate glycan structures, the sialylated S1H4N4F1 and the multisialylated S2H6N5 and S2H7N6F1 structures showed slightly higher relative abundance in the MSI stage IV samples than in the stage II samples (Supplementary Table S7).

#### 3.2.5. N-Glycan Profiles between Stage II and Stage IV in BRAFwt CRC

When the MSI BRAFwt II samples were compared to corresponding stage IV samples, only minor relative changes were observed in the neutral or acidic N-glycan profiles. In the neutral N-glycan profiles, only a few separate monosaccharide compositions showed significant differences, e.g., fucosylated hybrid-type structures H2N3F1 and H3N3F1 were more abundant in BRAFwt stage IV than in the stage II samples (Supplementary Table S6). In the acidic N-glycan profiles, a higher abundance of biantennary-size complex type glycans, but a clearly lower abundance of large-N-glycans (especially 5 HexNAc and 7 HexNAc or larger) was seen in the stage IV as compared to stage II samples (Table 6).

#### 3.2.6. N-Glycan Profiles between Stage II and Stage IV in BRAFmut CRC

Between the MSI BRAFmut stage II and IV samples, neutral N-glycan profiles similarly showed only minor relative changes, with monoantennary hybrid-type glycans and the fucosylated hybrid-type composition H2N3F1 being less abundant in BRAFmut stage IV than in stage II samples (Table 7 and Supplementary Table S6). More versatile relative differences were detected in the acidic N-glycan profiles of these MSI subgroups. In the BRAFmut stage IV samples, large N-glycans, putative terminal HexNAc containing glycans (especially in complex-type and bisecting sized complex-type glycans), and sulfated/phosphorylated complex-type glycans were significantly more abundant than in stage II samples, whereas 4 HexNAc, biantennary-size complex-type and sialylated complex-type glycans (especially S2H5N4) were relatively less abundant in BRAFmut stage IV than in stage II (Table 6 and Supplementary Table S7). Here it should be noted that the BRAFmut stage II MSI samples completely lacked the sulfated/phosphorylated N-glycans.

#### 3.2.7. N-Glycan Profiles between BRAFwt and BRAFmut Stage IV MSI Samples

**Figure 2:**
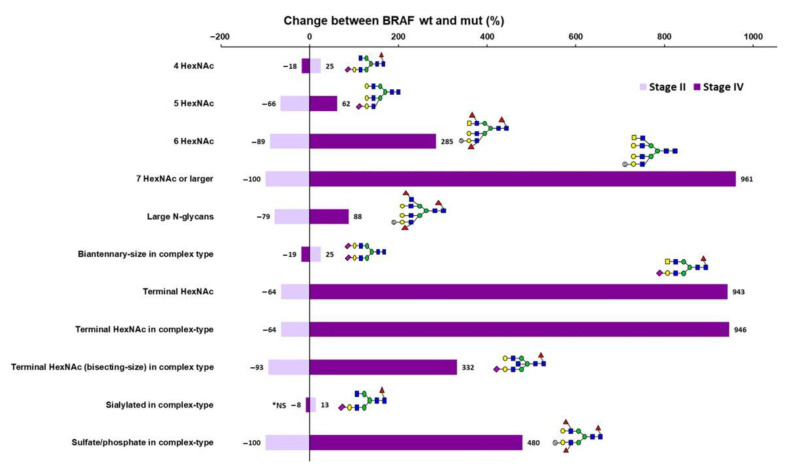
Change in the relative abundances of acidic N-glycan classes when comparing MSI BRAF^V600E^ wild-type and mutant CRC samples within the stage. The *x*-axis shows the percentage increase or decrease in the relative abundances of the significantly different acidic N-glycan classes between the BRAF wild-type and mutant samples within stage II (lavender) and stage IV (purple). Representative N-glycan structures are depicted by green circles (D-mannose), blue squares (N-acetyl-D-glucosamine), yellow squares (N-acetyl-D-galactosamine), red triangles (L-fucose), yellow circles (D-galactose), purple diamonds (N-acetylneuraminic acid, sialic acid), and open circles with an “S” (sulfate or phosphate ester). * NS = not significant (in stage IV).

Finally, when comparing the neutral N-glycan profiles between MSI stage IV BRAFwt and BRAFmut samples, only fucosylated pauci-mannose glycans and the composition H2N3F1 (fucosylated hybrid-type glycan) were significantly different, being less abundant in BRAFmut stage IV samples (Table 7 and Supplementary Table S6). The acidic profiles differed more significantly, and in direct contrast to the differences between MSI Stage II BRAFwt and BRAFmut samples, the MSI stage IV BRAFwt and BRAFmut samples showed differences in the same glycan classes, but in the opposite direction (Figure 2). The stage IV BRAFmut samples thus showed higher relative abundances of large N-glycans and putative terminal HexNAc structures (especially in complex-type and bisecting-size complex-type glycans), as well as sulfated/phosphorylated complex-type glycans, but fewer 4 HexNAc and biantennary-size complex-type glycans than did the BRAFwt stage IV samples (Table 6). The sialylated glycans were an exception, as those classes did not show significant differences between the stage IV subgroups. Of the monosaccharide compositions, the sialylated complex-type structures S1H5N5F1 and S1H6N5 showed a slight increase in relative abundance, and S1H4N4F1 showed a decrease in stage IV BRAFmut when compared to the corresponding BRAFwt samples (Supplementary Table S7).

## 4. Discussion

In this study, we investigated the N-linked glycan profiles of MSI CRC tissue specimens subdivided into subgroups according to stage (II or IV) and BRAF^V600E^ mutation status (wt or mut) using MALDI-TOF mass spectrometry. Further, we compared these glycan profiles to those of both paired non-neoplastic colon tissue samples and MSS CRC specimens. We found multiple differences between the MSI CRC samples and the control samples, and between the MSI stage II and MSS stage II CRC samples. When the MSI CRC subgroups were compared to each other, only minor differences were found in neutral N-glycan profiles, whereas a clear association between tumor stage and BRAF mutation status was observed in the acidic N-glycan profiles. Most interestingly, no acid ester-modified (sulfated/phosphorylated) N-glycans were identified in any of the stage II MSI tumors with the BRAF^V600E^ mutation.

In line with previous glycomic profiling reports of CRC tissues [17,18,19], MSI CRC tumors showed a higher relative abundance of neutral pauci-mannose N-glycans, especially the fucosylated glycan H2N2F1, and a decreased relative abundances of the putative terminal HexNAc (e.g., H3N5, H3N5F1, and H4N5F1) containing glycans, as well as the bisecting-size structure H5N5F1, as compared to the control tissues. In contrast to previous CRC reports, which have not specifically taken into account the MSI/MSS status [29,30,31,32], MSI tumors exhibited a lower relative abundance of high-mannose type N-glycans than did the non-neoplastic control tissues. Boyaval et al. [32] demonstrated even higher levels of high-mannose type N-glycans in the dysplastic regions of pre-cursor lesions than in early-stage CRC. Notably, in a recent study, the overexpression of high-mannose N-glycans was demonstrated, specifically in MSS CRC tumor tissue [33]. The acidic N-glycan profiles of MSI tumors were relatively simple, and, in contrast to the results of previous reports, MSI tumors showed a decreased relative abundances of some sulfated/phosphorylated and complex fucosylated structures, e.g., H5N4F3P1 and H5N6F4P1, as compared to the control tissues [17,19]. Fucosylated neutral pauci-mannose and hybrid-type glycans were detected to increase, while fucosylated acidic complex-type glycans were observed to decrease, in the MSI CRC samples compared to the non-neoplastic controls. Similarly, a higher abundance of fucosylated neutral N-glycans has been reported by Holm et al. [19] and a lower abundance of fucosylated complex-type N-glycans by Boyaval et al. [31] in CRC compared to the results for adjacent normal colon epithelium. Further, we did not find any statistically significant differences in the relative abundances of sialylated N-glycans between the MSI CRC samples and the control tissues, whereas previous reports have reported increased levels of sialylation in CRC [17,29,31,34], and increased sialylation has also been attributed to metastatic potential and therapeutic resistance in CRC [35,36].

When comparing BRAFwt stage II MSI and MSS tumors, multiple significant differences were observed, both in the neutral and acidic N-glycan profiles. The MSI tumors showed distinctively higher relative abundances of neutral complex-type and monoantennary hybrid-type glycans, as well as fucosylation, especially in pauci-mannose glycans. On the contrary, a clearly lower abundance of 2 HexNAc and high-mannose type glycans, as well as putative terminal HexNAc complex-type structures, was observed in the MSI than in MSS stage II tumors. In the acidic N-glycan profiles, MSI stage II BRAFwt tumors showed increased relative abundances of biantennary-size complex-type N-glycans and 4 HexNAc complex-type glycans than did the corresponding MSS samples. However, sulfated/phosphorylated hybrid-type glycans, large glycans, and putative terminal HexNAc containing complex-type glycans, as well as fucosylated, especially fucosylated/complex fucosylated complex-type glycans, were significantly less abundant in the MSI stage II BRAFwt tumors. These neutral and acidic N-glycan types, with differing abundances in MSI stage II CRC compared to corresponding MSS tumors, may be linked to the MSI pathway of CRC. To our knowledge, this is the first study to report significant N-glycosylation differences between MSI and MSS CRC tissue samples.

Between MSI CRC subgroups, only minor differences were found in neutral N-glycan profiles, and the major differences were observed in the acidic N-glycan profiles. Most interestingly, no sulfated/phosphorylated N-glycans were identified in any of the stage II MSI tumors containing the BRAF^V600E^ mutation. Between all MSI stage II and stage IV CRC, only acidic biantennary-size complex-type glycans showed a clear decrease in the stage IV samples. When comparing MSI BRAFwt stage II and IV CRC samples to each other, biantennary-size complex type glycans were more abundant, and larger N-glycans were less abundant in stage IV samples. Between the BRAFmut stage II and stage IV samples, large N-glycans, putative terminal HexNAc containing, and sulfated/phosphorylated complex-type glycans were significantly more abundant, and 4 HexNAc, biantennary-size complex-type and sialylated complex-type glycans were less abundant in the stage BRAFmut IV samples. The most interesting differences were observed when comparing the MSI BRAFwt and BRAFmut samples within stages. In these comparisons, the same glycan classes differed between the BRAFwt and BRAFmut samples, but the direction of the change was totally opposite in stage II versus stage IV. In the stage II BRAFmut samples, sulfated, large, and putative terminal HexNAc containing acidic N-glycans were decreased when compared to the corresponding BRAFwt samples, whereas in stage IV, these same N-glycan features were increased in the BRAFmut as compared to the BRAFwt samples. On the other hand, increased levels of biantennary-size complex-type glycans, especially 4 HexNAc glycans, were observed in the stage II BRAFmut samples as compared to the corresponding BRAFwt samples, whereas in the stage IV samples, these same glycan classes were decreased in the BRAFmut relative to the BRAFwt samples. The *BRAF*^V600E^ mutation is known to have a negative prognostic value in MSS CRC, while MSI has been suggested to override this negative effect [3,4,6]. Some studies have reported that the *BRAF* mutation could be a positive prognostic marker in early stage MSI CRC [37], but a negative prognostic factor in advanced MSI CRC [38,39]. The oppositely behaving acidic N-glycan types, especially large, sulfated/phosphorylated, and putative terminal HexNAc containing glycans, showed a clear dependence on tumor stage and BRAF mutation status and may thus be associated with MSI CRC progression according to BRAF mutation.

Large N-glycans commonly contain β1,6-branching, and an increase in β1,6-branched N-linked glycans has been related to malignant transformation and metastatic potential in many cancers, including CRC [13,40,41,42]. Importantly, the modification of epithelial cadherin (E-cadherin) with branched glycans is known to interfere with cellular adhesion and promote tumor invasiveness and metastasis [13,43]. Also, Kaprio et al. [20] reported a significantly higher abundance of acidic N5 glycans in tissue from stage III CRC compared to stage I–II CRC samples. The enzyme catalyzing β1,6-branching of N-glycans is N-acetylglucosaminyltransferase V (GnT-V), encoded by the *MGAT5* gene. *MGAT5* expression is regulated by the RAS–RAF–MAPK pathway, and mutations of this oncogenic pathway are known to upregulate GnT-V expression and concomitant β1,6-branching [13]. This is in line with our findings showing increased levels of large (N ≥ 5) acidic N-glycans in BRAFmut stage IV as compared to those of the corresponding BRAFwt tumors but contradicts with the findings that BRAFmut stage II tumors show lower levels of these glycans as compared to those of the corresponding BRAFwt samples. Also, in our study, BRAFwt MSI stage II samples showed a lower relative abundance of large acidic N-glycans than did the BRAFwt stage II MSS tumors, thus potentially explaining, to some degree, the better prognosis of early stage MSI CRC as compared to corresponding MSS tumors.

Sulfated glycans have been shown to play an important role in many cell surface-related functions, such as cellular adhesion and selectin–ligand interactions [44]. Higher levels of glycan-sulfotransferase activities have been demonstrated in poorly differentiated gastric carcinomas than in moderately differentiated tumors, thus being associated with gastric tumorigenesis [45]. Moreover, sulfated Lewis X determinants form a predominant structural glycan motif in the xenograft tumor mucin of LS174T-HM7 cells, a highly metastatic subline of the LS174T human CRC cell line [46]. Sulfated glycans are preferably bound by galectin-1 and galectin-2 [47], and the upregulation of galectin-1 has been related to malignant progression in CRC [48,49]. In our study, sulfated/phosphorylated complex-type glycans were increased with the tumor stage in the MSI BRAFmut, but not in the MSI BRAFwt, samples. Strikingly, sulfated/phosphorylated N-glycans were not found in any of the stage II MSI BRAFmut tumors, whereas MSI BRAFwt stage II tumors displayed these glycans. Further, MSI stage II BRAFwt tumors showed a lower relative abundance of sulfated/phosphorylated hybrid-type glycans when compared to the corresponding MSS samples.

Putative terminal HexNAc-containing N-glycans (N > H > 1) have previously been identified in various CRC cell lines, and an increased abundance of terminal HexNAc residues has further been correlated with caudal-related homeobox protein 1 (CDX1) expressing CRC cells [50,51]. CDX1 is a transcription factor regulating the normal development and differentiation of the intestinal epithelium and is associated with tumor suppressing potential in the colon [52]. More specifically, increased terminal N-acetylglucosamine (GlcNAc) has been identified in various carcinomas [22]. N-glycans containing bisecting GlcNAc have been more significantly attributed to the suppression of tumor progression and metastasis through the regulation of cell surface glycoproteins, such as stabilizing the E-cadherin mediated cell–cell adhesion [13,43]. N-glycans containing bisecting GlcNAc have also been reported to decrease in CRC tissue samples with more advanced tumor stages [30,34]. In contrast, in our study, stage IV BRAFmut tumors showed a higher relative abundance of acidic putative terminal HexNAc, especially in bisecting-size, containing complex-type glycans when compared to corresponding stage II samples. However, a higher expression of bisecting N-glycans in a metastatic MSI CRC cell line (LIM1215) as compared with two non-metastatic MSI CRC cell lines (LIM1899 and LIM2405) has also been controversially demonstrated by Sethi et al. [53]. Further, the acidic putative terminal HexNAc and terminal HexNac in bisecting-size complex-type glycans showed a significantly lower relative abundance in MSI stage II than in corresponding MSS samples.

A limitation of our study is that the mass spectrometric analyses were conducted using flakes from FFPE tissue blocks. Thus, it is not possible to specify from which cell type (e.g., cancer or stromal cells) the detached glycans originated. The tumor stroma is composed of various non-neoplastic cells, e.g., immune cells, fibroblasts, and endothelial cells, as well as the extracellular matrix that forms a tumor microenvironment promoting cancer growth and spreading [54]. Moreover, the cancer related N-glycan signature has been demonstrated to spread into the stroma at the invasive front of the tumor [31]. We, however, used macrodissection to exclude the distant stroma and to achieve the highest percentage of epithelial cells in the carcinoma and non-neoplastic tissues. The analyzed tissues (tumor epithelium percentages 30–80%), however, included varying amounts of tumor mucin, intra-tumor stroma, and surrounding interface stroma, which may contribute to the heterogeneity of the N-glycan signatures found in this study. An additional limitation of this study is that we used pools of paired non-neoplastic colon samples from each MSI CRC patient set, instead of individual paired non-neoplastic control samples. However, the main aim of this study was to compare the N-glycan profiles between MSI and MSS CRC samples, specifically between different MSI CRC subgroups.

## 5. Conclusions

Our study identified multiple differences in N-glycan profiles between the MSI tumors and the control tissues, the stage II MSI and MSS CRC samples, and within different MSI subgroups. Most importantly, we demonstrated that molecular subgroups of MSI CRCs exhibit distinct glycan profiles that may explain certain carcinogenic characteristics of these CRC subtypes. After further characterization and validation, these N-glycan features may provide new biomarkers to refine the prognosis of MSI CRC tumors. Further, immunotherapy strategies targeting cancer-associated glycan features may be potential therapeutic agents [55]. Here, the identification of the carrier glycoproteins could also open new possibilities for specific diagnostics and targeted therapies by exploiting glycan-protein epitopes.

## Figures and Tables

**Table 1:** Seven different study groups analyzed for neutral and acidic N-glycans.

Number	Study Group	*n*
1	Non-neoplastic controls vs. all MSI CRC	4 pools vs. 40
2	CRC Stage II BRAFwt MSI vs. MSS	10 vs. 9
3	MSI CRC Stage II BRAFwt vs. BRAFmut	10 vs. 10
4	MSI CRC Stage II vs. IV	20 vs. 20
5	MSI CRC BRAFwt Stage II vs. IV	10 vs. 10
6	MSI CRC BRAFmut Stage II vs. IV	10 vs. 10
7	MSI CRC Stage IV BRAFwt vs. BRAFmut	10 vs. 10

**Table 2:** Characteristics of the CRC subgroups used in the study.

Subgroup			Subgroup		
**All MSI CRC (*n* = 40)**	Age	73 (51–89)	**MSS CRC Stage II BRAFwt (*n* = 9)**	Age	59 (52–77)
	Sex	♀ 28 (70%)		Sex	♀ 4 (44%)
		♂ 12 (30%)			♂ 5 (56%)
	Tumor site	R 35 (88%)		Tumor site	R 4 (44%)
		L 5 (12%)			L 5 (56%)
	Grade	LG 23 (58%)		Grade	LG 7 (78%)
		HG 17 (42%)			HG 2 (22%)
	pT	T2 1 (2%)		pT	T2 0 (0%)
		T3 25 (63%)			T3 8 (89%)
		T4 14 (35%)			T4 1 (11%)
	pN	N0 26 (65%)		pN	N0 9 (100%)
		N1 3 (7%)			N1 0 (0%)
		N2 11 (28%)			N2 0 (0%)
	M	M0 20 (50%)		M	M0 9 (100%)
		M1 20 (50%)			M1 0 (0%)
**MSI CRC Stage II BRAFwt (*n* = 10)**	Age	72 (51–78)	**MSI CRC Stage II BRAFmut (*n* = 10)**	Age	71 (58–82)
	Sex	♀ 7 (70%)		Sex	♀ 7 (70%)
		♂ 3 (30%)			♂ 3 (30%)
	Tumor site	R 8 (80%)		Tumor site	R 8 (80%)
		L 2 (20%)			L 2 (20%)
	Grade	LG 6 (60%)		Grade	LG 6 (60%)
		HG 4 (40%)			HG 4 (40%)
	pT	T3 7 (70%)		pT	T3 8 (80%)
		T4 3 (30%)			T4 2 (20%)
	pN	N0 10 (100%)		pN	N0 10 (100%)
	M	M0 10 (100%)		M	M0 10 (100%)
**MSI CRC Stage IV BRAFwt (*n* = 10)**	Age	74 (54–86)	**MSI CRC Stage IV BRAFmut (*n* = 10)**	Age	75 (63–89)
	Sex	♀ 7 (70%)		Sex	♀ 7 (70%)
		♂ 3 (30%)			♂ 3 (30%)
	Tumor site	R 9 (90%)		Tumor site	R 10 (100%)
		L 1 (10%)			L 0 (0%)
	Grade	LG 4 (40%)		Grade	LG 7 (70%)
		HG 6 (60%)			HG 3 (30%)
	pT	T2 1 (10%)		pT	T2 0 (0%)
		T3 3 (30%)			T3 7 (79%)
		T4 6 (60%)			T4 3 (30%)
	pN	N0 1 (10%)		pN	N0 5 (50%)
		N1 3 (30%)			N1 0 (0%)
		N2 6 (60%)			N2 5 (50%)
	M	M1a 4 (40%)		M	M1a 5 (50%)
		M1b 2 (20%)			M1b 4 (40%)
		M1c 4 (40%)			M1c 1 (10%)

CRC, colorectal cancer; HG, high-grade; L, left; LG, low-grade; M, metastases; mut, mutated; N, nodes; MSI, microsatellite instability; MSS, microsatellite stable; R, right; T, tumor; wt, wild-type.

**Table 3:** Relative abundances of significantly different neutral and acidic N-glycan classes between the controls (*n* = 4 pools) and the MSI CRC samples (*n* = 40).

	Ctrls		MSI CRC		Fold Change	*p*-Adjusted ^a^
	Average (%)	SEM	Average (%)	SEM		
**NEUTRAL**						
5 HexNAc	15.3	1.3	8.3	0.7	0.5	0.007
High-mannose type	40.2	2.6	33.0	1.2	0.8	0.039
Pauci-mannose type	15.5	1.3	24.1	1.2	1.6	0.045
Biantennary-size in complex-type	25.7	1.7	36.8	1.6	1.4	0.044
Monoantennary in hybrid-type	38.0	1.8	45.2	1.0	1.2	0.028
Fucosylation in pauci-mannose type	51.7	1.3	71.8	1.8	1.4	0.011
Fucosylation in hybrid-type	52.2	1.2	65.3	2.0	1.3	0.014
Terminal HexNAc	17.7	1.3	13.1	1.0	0.7	0.039
**ACIDIC**						
Fucosylation in complex-type	71.9	1.9	57.8	2.0	0.8	0.028

^a^ Benjamini–Hochberg; CRC, colorectal cancer; HexNAc, N-acetylhexosamine; MSI, microsatellite instability; SEM, standard error of mean.

**Table 4:** Relative abundances of significantly different neutral N-glycan classes between MSS BRAFwt stage II and MSI BRAFwt stage II samples.

	MSS St II BRAFwt		MSI St II BRAFwt		Fold Change	*p*-Adjusted ^a^
	Average (%)	SEM	Average (%)	SEM		
2 HexNAc	74.1	3.0	54.8	5.0	0.7	0.005
3 HexNAc	6.4	0.4	4.8	0.3	0.8	0.016
4 HexNAc	9.5	1.2	29.1	4.7	3.1	0.001
5 HexNAc	4.6	1.0	8.5	1.4	1.8	0.030
High-mannose type	53.8	2.4	31.9	3.2	0.6	0.001
Complex type	16.8	2.5	40.4	4.9	2.4	0.002
Biantennary-size in complex-type	27.5	4.1	41.1	3.4	1.5	0.025
Monoantennary in hybrid-type	29.3	4.2	43.1	2.8	1.5	0.013
Fucosylation	25.6	2.3	50.5	5.1	2.0	0.006
Fucosylation in pauci-mannose type	43.0	3.0	69.9	5.5	1.6	0.005
Terminal HexNAc in hybrid-type	1.5	1.0	3.1	0.3	2.1	0.040
Terminal HexNAc in complex-type	41.7	5.1	27.9	2.8	0.7	0.030

^a^ Benjamini–Hochberg HexNAc, N-acetylhexosamine; MSI, microsatellite instability; MSS, microsatellite stabile; mut, mutated; SEM, standard error of mean; St, stage; wt, wild-type.

**Table 5:** Relative abundances of significantly different acidic N-glycan classes between MSS BRAFwt stage II and MSI BRAFwt stage II samples.

	MSS St II BRAFwt		MSI St II BRAFwt		Fold Change	*p*-Adjusted ^a^
	Average (%)	SEM	Average (%)	SEM		
3 HexNAc	10.0	1.3	1.9	0.5	0.2	0.002
4 HexNAc	41.9	4.3	74.6	3.6	1.8	0.002
5 HexNAc	19.8	1.0	13.8	0.8	0.7	0.001
6 HexNAc	14.9	1.9	5.4	1.5	0.4	0.007
7 HexNAc or larger	13.5	3.4	4.3	1.6	0.3	0.030
Complex-type	90.1	1.3	98.1	0.5	1.1	0.002
Biantennary-size in complex-type	42.9	4.7	75.5	3.5	1.8	0.002
Fucosylation	75.6	2.9	56.3	4.3	0.7	0.017
Fucosylation in complex-type	76.8	3.0	55.8	4.2	0.7	0.013
Complex fucosylation	35.0	5.2	12.6	2.6	0.4	0.011
Complex fucosylation in hybrid-type	16.1	5.2	1.5	1.5	0.1	0.011
Complex fucosylation in complex-type	36.9	5.3	12.8	2.7	0.3	0.011
Terminal HexNAc	20.9	5.4	2.0	0.8	0.1	0.004
Terminal HexNAc in complex-type	23.3	6.0	2.0	0.8	0.1	0.003
Terminal HexNAc (bisecting-size) in complex-type	14.0	2.8	5.3	2.0	0.4	0.017
Sulfate/phosphate in hybrid type	34.7	8.6	9.4	6.3	0.3	0.019

^a^ Benjamini–Hochberg HexNAc, N-acetylhexosamine; MSI, microsatellite instability; MSS, microsatellite stabile; mut, mutated; SEM, standard error of mean; St, stage; wt, wild-type.

**Table 6:** Relative abundances of significantly different acidic N-glycan classes between MSI CRC subgroups.

	MSI St II BRAFwt		MSI St II BRAFmut		Fold Change	*p*-Adjusted ^a^
	Average (%)	SEM	Average (%)	SEM		
4 HexNAc	74.6	3.6	93.2	1.2	1.2	0.002
5 HexNAc	13.8	0.8	4.7	0.6	0.3	0.001
6 HexNAc	5.4	1.5	0.6	0.2	0.1	0.007
7 HexNAc or larger	4.3	1.6	0.0	0.0	None in BRAFmut	0.001
Large N-glycans	21.9	2.9	4.7	0.7	0.2	0.001
Biantennary-size in complex-type	75.5	3.5	94.4	0.7	1.3	0.001
Terminal HexNAc	2.0	0.8	0.7	0.7	0.4	0.023
Terminal HexNAc in complex-type	2.0	0.8	0.7	0.7	0.4	0.023
Terminal HexNAc (bisecting-size) in complex-type	5.3	2.0	0.4	0.2	0.1	0.005
Sialylated	88.1	4.6	100.0	0.0	1.1	0.008
Sialylated complex-type	88.1	4.6	100.0	0.0	1.1	0.008
Sulfate or phosphate	12.0	4.6	0.0	0.0	None in BRAFmut	0.008
Sulfate/phosphate in complex-type	12.0	4.6	0.0	0.0	None in BRAFmut	0.008
	**MSI St II**		**MSI St IV**		**Fold Change**	***p*-Adjusted ^a^**
	**Average (%)**	**SEM**	**Average (%)**	**SEM**		
Biantennary-size in complex-type	84.9	2.8	78.0	2.9	0.9	0.038
	**MSI St II BRAFwt**		**MSI St IV BRAFwt**		**Fold Change**	***p*-Adjusted ^a^**
	**Average (%)**	**SEM**	**Average (%)**	**SEM**		
5 HexNAc	13.8	0.8	10.2	0.4	0.7	0.009
7 HexNAc or larger	4.3	1.6	0.4	0.2	0.1	0.030
Large N-glycans	21.9	2.9	12.3	0.7	0.6	0.020
Biantennary-size in complex-type	75.5	3.5	86.0	0.8	1.1	0.032
	**MSI St II BRAFmut**		**MSI St IV BRAFmut**		**Fold Change**	***p*-Adjusted ^a^**
	**Average (%)**	**SEM**	**Average (%)**	**SEM**		
4 HexNAc	93.2	1.2	69.7	4.3	0.7	0.001
5 HexNAc	4.7	0.6	16.5	1.6	3.5	0.001
6 HexNAc	0.6	0.2	7.7	1.9	12.8	0.002
7 HexNAc or larger	0.0	0.0	4.5	1.6	None in St II	0.001
Large N-glycans	4.7	0.7	23.1	3.1	4.9	0.001
Biantennary-size in complex-type	94.4	0.7	69.9	4.5	0.7	0.001
Terminal HexNAc	0.7	0.7	6.1	1.8	8.7	0.006
Terminal HexNAc in complex-type	0.7	0.7	6.2	1.8	8.9	0.006
Terminal HexNAc (bisecting-size) in complex-type	0.4	0.2	7.9	3.4	19.8	0.001
Sialylated	100.0	0.0	89.8	3.4	0.9	0.004
Sialylated complex-type	100.0	0.0	89.9	3.4	0.9	0.004
Sulfate/phosphate	0.0	0.0	10.7	3.4	None in St II	0.002
Sulfate/phosphate in complex-type	0.0	0.0	10.6	3.4	None in St II	0.002
	**MSI St IV BRAFwt**		**MSI St IV BRAFmut**		**Fold Change**	***p*-Adjusted ^a^**
	**Average (%)**	**SEM**	**Average (%)**	**SEM**		
4 HexNAc	85.2	0.8	69.7	4.3	0.8	0.002
5 HexNAc	10.2	0.4	16.5	1.6	1.6	0.005
6 HexNAc	2.0	0.6	7.7	1.9	3.9	0.005
7 HexNAc or larger	0.4	0.2	4.5	1.6	11.3	0.011
Large N-glycans	12.3	0.7	23.1	3.1	1.9	0.001
Biantennary-size in complex-type	86.0	0.8	69.9	4.5	0.8	0.001
Terminal HexNAc	0.6	0.2	6.1	1.8	10.2	0.019
Terminal HexNAc in complex-type	0.6	0.2	6.2	1.8	10.3	0.019
Terminal HexNAc (bisecting-size) in complex-type	1.8	0.5	7.9	3.4	4.4	0.007
Sulfate/phosphate	1.8	1.7	10.7	3.4	5.9	0.019
Sulfate/phosphate in complex-type	1.8	1.7	10.6	3.4	5.9	0.019

^a^ Benjamini–Hochberg HexNAc, N-acetylhexosamine; MSI, microsatellite instability; mut, mutated; SEM, standard error of mean; St, stage; wt, wild-type.

**Table 7:** Relative abundances of significantly different neutral N-glycan classes between MSI subgroups.

	MSI St II		MSI St IV		Fold Change	*p*-Adjusted ^a^
	Average (%)	SEM	Average (%)	SEM		
3 HexNAc	5.3	0.3	6.7	0.4	1.3	0.017
Fucosylation in high-mannose type	0.9	0.1	1.1	0.1	1.2	0.027
	**MSI St II BRAFmut**		**MSI St IV BRAFmut**		**Fold Change**	***p*-Adjusted ^a^**
	**Average (%)**	**SEM**	**Average (%)**	**SEM**		
Monoantennary in hybrid-type	49.5	1.1	41.6	1.3	0.8	0.008
	**MSI St IV BRAFwt**		**MSI St IV BRAFmut**		**Fold Change**	***p*-Adjusted ^a^**
	**Average (%)**	**SEM**	**Average (%)**	**SEM**		
Fucosylation in pauci-mannose type	77.3	1.2	66.0	3.9	0.9	0.024

^a^ Benjamini–Hochberg HexNAc, N-acetylhexosamine; MSI, microsatellite instability; mut, mutated; SEM, standard error of mean; St, stage; wt, wild-type.

## Data Availability

Data is contained within the article or Supplementary Materials.

## Supplementary Materials

The following supporting information can be downloaded at: https://www.mdpi.com/article/10.3390/cancers15143571/s1, Figure S1: Representative unprocessed MALDI-TOF mass spectra of (A) neutral and (B) acidic N-linked glycans isolated from a tumor tissue sample from a patient with stage II MSI BRAF^V600E^ wild-type colon cancer. Note the different scales on the y-axes; Table S1: MS data of acidic N-glycans; Table S2: MS data of neutral N-glycans; Table S3: Significantly different neutral and acidic monosaccharide compositions between controls (*n* = 4 pools) and MSI CRC samples (*n* = 40); Table S4: Significantly different neutral monosaccharide compositions between MSS BRAFwt stage II and MSI BRAFwt stage II samples; Table S5: Significantly different acidic monosaccharide compositions between MSS BRAFwt stage II and MSI BRAFwt stage II samples; Supplementary Table S6: Significantly different neutral monosaccharide compositions between MSI subgroups; Table S7: Significantly different acidic monosaccharide compositions between MSI subgroups.
