# Supplementary material for: N-Glycomic Profiling of Microsatellite Unstable Colorectal Cancer
Source: Cancers (Basel). 2023 Jul 11;15(14):3571. doi: 10.3390/cancers15143571 (PMC10376987; doi:10.3390/cancers15143571)
Supplement: Supplementary file 1 [file cancers-15-03571-s001.zip › Supplementary Material Ukkola.pdf]

## Supplementary Information

for

# N-Glycomic Profiling of Microsatellite Unstable Colorectal Cancer

Iiris Ukkola[1,2], Pirjo Nummela[1,2], Annamari Heiskanen[3], Matilda Holm[1,2,4,5†], Sadia Zafar[1,2], Mia Kero[1], Caj Haglund[4,5] Tero Satomaa[3], Soili Kytölä[6], and Ari Ristimäki[1,2*]

[1]Department of Pathology, HUSLAB, HUS Diagnostic Center, Helsinki University Hospital and University of Helsinki, Helsinki, Finland

[2]Applied Tumor Genomics Research Program, Research Programs Unit, University of Helsinki and Helsinki University Hospital, Helsinki, Finland

[3]Glykos Finland Ltd., Helsinki, Finland

[4]Translational Cancer Medicine Research Program, Faculty of Medicine, University of Helsinki, Helsinki, Finland

[5]Department of Surgery, Helsinki University Hospital and University of Helsinki, Helsinki, Finland

[6]Department of Genetics, HUSLAB, HUS Diagnostic Center, Helsinki University Hospital and University of Helsinki, Helsinki, Finland

[†]**Current address:** Science for Life Laboratory, Department of Protein Science, KTH Royal Institute of Technology, Solna, Sweden and Department of Biosciences and Nutrition, Karolinska Institutet, Huddinge, Sweden

***Correspondence to** Ari Ristimäki, Pathology, HUSLAB, P.O. Box 400, FI-00029 HUS, Finland. E-mail: ari. ristimaki (at) helsinki. fi

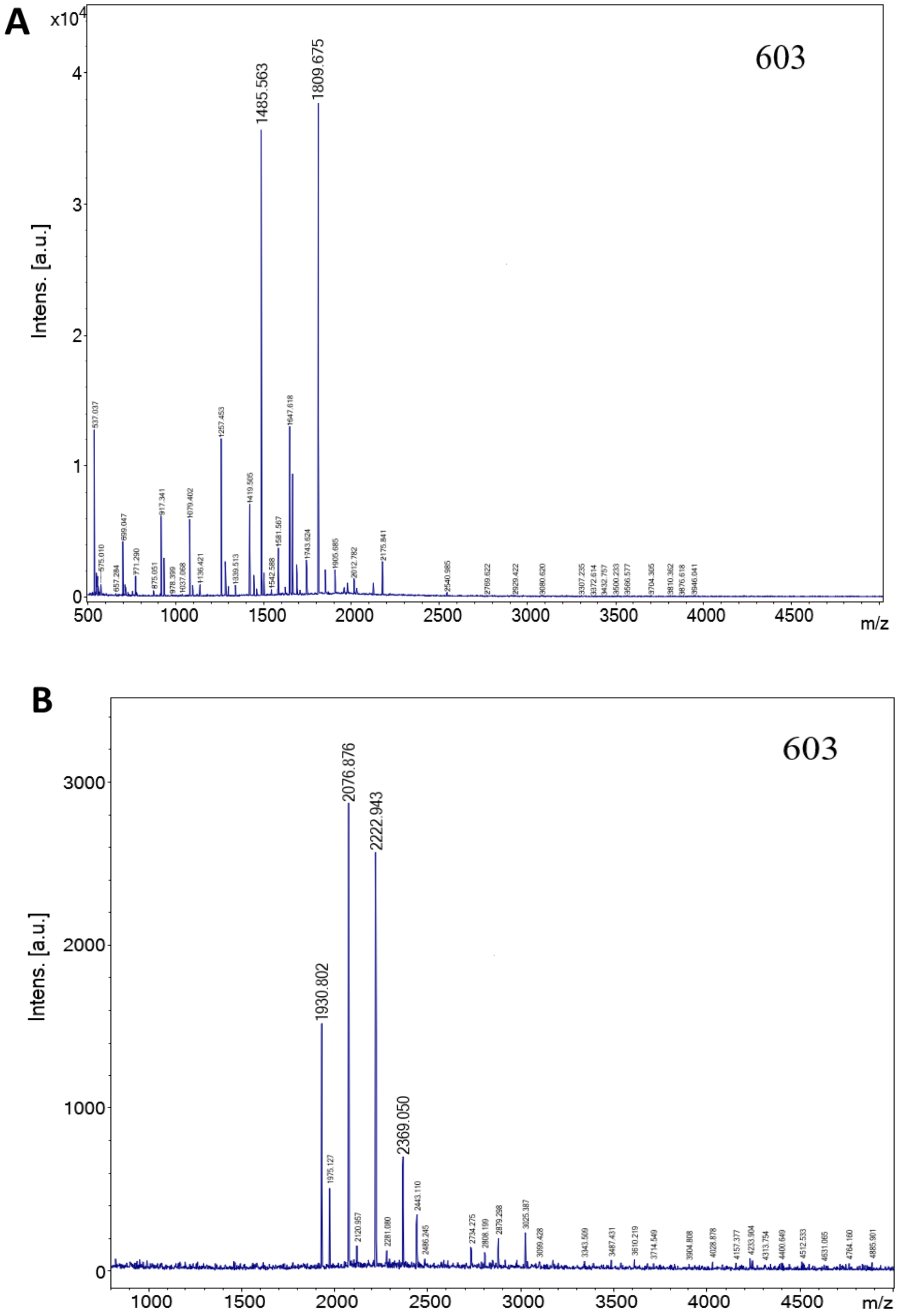


**Supplementary Figure S1.** Representative unprocessed MALDI-TOF mass spectra of (A) neutral and (B) acidic N-linked glycans isolated from a tumor tissue sample from a patient with stage II MSI BRAF$^{V600E}$ wild-type colon cancer. Note the different scales on y-axes.

**Supplementary Table S3.** Significantly different neutral and acidic monosaccharide compositions between controls (*n*=4 pools) and MSI CRC samples (*n*=40)

| | Ctrls | | MSI CRC | | | |
|---|---|---|---|---|---|---|
| | **Average (%)** | **SEM** | **Average (%)** | **SEM** | **Fold change** | ***p*-Adjusted**[a] |
| **NEUTRAL** | | | | | | |
| H6N2 | 11.3 | 0.7 | 8.9 | 0.4 | 0.8 | 0.032 |
| H9N2 | 6.7 | 0.5 | 4.4 | 0.3 | 0.7 | 0.032 |
| H10N2 | 0.2 | 0.0 | 0.1 | 0.0 | 0.5 | 0.008 |
| H1N2 | 0.1 | 0.0 | 0.3 | 0.0 | 3.0 | 0.028 |
| H3N2 | 3.6 | 0.3 | 2.6 | 0.2 | 0.7 | 0.043 |
| H1N2F1 | 0.1 | 0.0 | 0.9 | 0.1 | 9.0 | 0.005 |
| H2N2F1 | 3.4 | 0.4 | 11.7 | 0.9 | 3.4 | 0.006 |
| H2N3F1 | 0.1 | 0.0 | 0.2 | 0.0 | 2.0 | 0.013 |
| H3N3F1 | 0.9 | 0.1 | 1.8 | 0.1 | 2.0 | 0.013 |
| H4N3F1 | 0.6 | 0.0 | 1.1 | 0.1 | 1.8 | 0.025 |
| H4N3F2 | 0.1 | 0.0 | 0.3 | 0.0 | 3.0 | 0.029 |
| H3N5 | 1.2 | 0.3 | 0.5 | 0.1 | 0.4 | 0.012 |
| H3N5F1 | 4.3 | 0.2 | 1.6 | 0.2 | 0.4 | 0.004 |
| H4N5F1 | 2.6 | 0.2 | 1.3 | 0.1 | 0.5 | 0.009 |
| H5N5F1 | 2.2 | 0.4 | 0.9 | 0.1 | 0.4 | 0.004 |
| H4N5F2 | 0.9 | 0.1 | 0.4 | 0.1 | 0.4 | 0.045 |
| H5N5F2 | 1.2 | 0.3 | 0.2 | 0.0 | 0.2 | 0.008 |
| H4N5F3 | 0.6 | 0.1 | 0.2 | 0.0 | 0.3 | 0.011 |
| **ACIDIC** | | | | | | |
| H5N4F3P1 | 1.0 | 0.4 | 0.3 | 0.1 | 0.3 | 0.025 |
| H4N5F3P1 | 1.6 | 0.7 | 0.1 | 0.0 | 0.1 | <0.001 |
| H5N5F2P1 | 1.4 | 0.7 | 0.0 | 0.0 | None in CRC | <0.001 |
| H5N5F3P1 | 1.3 | 0.6 | 0.1 | 0.0 | 0.1 | 0.002 |
| H5N6F4P1 | 0.6 | 0.3 | 0.1 | 0.1 | 0.2 | 0.001 |
| H6N6F3P1 | 1.1 | 0.5 | 0.1 | 0.0 | 0.1 | 0.004 |

[a]Benjamini-Hochberg; CRC, colorectal cancer; F, deoxyhexose; H, hexose; MSI, microsatellite instability; N, N-acetylhexosamine; P, acid ester; SEM, standard error of mean

**Supplementary Table S4.** Significantly different neutral monosaccharide compositions between MSS BRAFwt stage II and MSI BRAFwt stage II samples

| | MSS St II BRAFwt | | MSI St II BRAFwt | | | |
|---|---|---|---|---|---|---|
| | **Average (%)** | **SEM** | **Average (%)** | **SEM** | **Fold change** | ***p*-Adjusted**[a] |
| H5N2 | 15.5 | 1.3 | 8.5 | 0.8 | 0.5 | 0.001 |
| H6N2 | 12.5 | 0.7 | 8.1 | 0.8 | 0.6 | 0.003 |
| H7N2 | 8.1 | 0.7 | 4.9 | 0.5 | 0.6 | 0.004 |
| H8N2 | 8.8 | 0.6 | 5.5 | 0.8 | 0.6 | 0.006 |
| H10N2 | 0.4 | 0.1 | 0.1 | 0.1 | 0.3 | 0.011 |
| H2N2 | 4.3 | 0.3 | 2.4 | 0.3 | 0.6 | 0.005 |
| H3N2 | 5.0 | 0.5 | 2.6 | 0.5 | 0.5 | 0.008 |
| H4N2 | 2.7 | 0.2 | 0.9 | 0.1 | 0.3 | <0.001 |
| H3N3 | 0.8 | 0.1 | 0.5 | 0.1 | 0.6 | 0.025 |
| H5N3 | 0.7 | 0.1 | 0.3 | 0.0 | 0.4 | 0.002 |
| H6N3 | 0.7 | 0.1 | 0.4 | 0.0 | 0.6 | 0.020 |
| H2N2F1 | 5.4 | 0.8 | 11.4 | 2.4 | 2.1 | 0.037 |
| H4N2F1 | 0.3 | 0.0 | 0.2 | 0.0 | 0.7 | 0.024 |
| H2N3F1 | 0.0 | 0.0 | 0.1 | 0.0 | None in MSS | 0.003 |
| H5N3F1 | 0.4 | 0.1 | 0.1 | 0.0 | 0.3 | 0.003 |
| H6N3F1 | 0.4 | 0.1 | 0.1 | 0.0 | 0.3 | 0.014 |
| H3N4 | 0.7 | 0.1 | 0.5 | 0.1 | 0.7 | 0.020 |
| H5N4 | 1.6 | 0.3 | 4.0 | 0.8 | 2.5 | 0.016 |
| H3N4F1 | 1.6 | 0.2 | 6.5 | 1.9 | 4.1 | 0.002 |
| H4N4F1 | 1.2 | 0.1 | 4.1 | 0.8 | 3.4 | <0.001 |
| H5N4F1 | 1.7 | 0.2 | 11.5 | 2.0 | 6.8 | <0.001 |
| H5N5F1 | 0.3 | 0.1 | 0.8 | 0.1 | 2.7 | 0.005 |
| H6N5F1 | 0.3 | 0.0 | 1.4 | 0.3 | 4.7 | <0.001 |
| H7N6F1 | 0.0 | 0.0 | 0.3 | 0.1 | None in MSS | <0.001 |

[a]Benjamini-Hochberg; F, deoxyhexose; H, hexose; MSI, microsatellite instability; MSS, microsatellite stable; N, N-acetylhexosamine; St, stage; mut, mutated; wt, wild-type

**Supplementary Table S5.** Significantly different acidic monosaccharide compositions between MSS BRAFwt stage II and MSI BRAFwt stage II samples

| | **MSS St II BRAFwt** | | **MSI St II BRAFwt** | | | |
|---|---|---|---|---|---|---|
| | **Average (%)** | **SEM** | **Average (%** | **SEM** | **Fold change** | ***p*-Adjusted[a]** |
| S1H4N4F1 | 1.6 | 0.2 | 0.4 | 0.1 | 0.3 | 0.001 |
| S1H5N4F1 | 14.8 | 2.1 | 25.6 | 2.1 | 1.7 | 0.006 |
| S1H4N5F1 | 1.5 | 0.2 | 0.3 | 0.1 | 0.2 | <0.001 |
| S1H5N4F2 | 2.2 | 0.3 | 4.8 | 0.6 | 2.2 | 0.005 |
| S1H4N5F2 | 0.3 | 0.1 | 0.0 | 0.0 | None in MSI | 0.010 |
| S1H5N5F3 | 0.6 | 0.4 | 0.0 | 0.0 | None in MSI | 0.010 |
| S2H5N4 | 4.7 | 0.7 | 18.0 | 1.7 | 3.8 | <0.001 |
| S2H5N4F1 | 3.1 | 0.7 | 7.4 | 1.1 | 2.4 | 0.005 |
| S2H6N5F3 | 0.1 | 0.1 | 0.6 | 0.2 | 6.0 | 0.027 |
| S3H6N5 | 0.1 | 0.1 | 1.2 | 0.2 | 12.0 | 0.002 |
| S3H6N5F1 | 0.1 | 0.1 | 1.1 | 0.2 | 11.0 | 0.001 |
| H4N5F3P1 | 0.4 | 0.1 | 0.1 | 0.1 | 0.3 | 0.018 |
| H5N5F2P1 | 0.7 | 0.3 | 0.0 | 0.0 | None in MSI | 0.026 |
| H5N6F2P1 | 0.9 | 0.3 | 0.0 | 0.0 | None in MSI | 0.002 |
| H5N6F3P1 | 1.2 | 0.3 | 0.1 | 0.1 | 0.1 | 0.002 |
| H6N6F3P1 | 0.7 | 0.3 | 0.1 | 0.1 | 0.1 | 0.041 |

[a]Benjamini-Hochberg; F, deoxyhexose; H, hexose; MSI, microsatellite instability; MSS, microsatellite stable; N, N-acetylhexosamine; St, stage; mut, mutated; wt, wild-type

**Supplementary Table S6.** Significantly different neutral monosaccharide compositions between MSI subgroups

| | **MSI St II BRAFwt** | | **MSI St II BRAFmut** | | | |
|---|---|---|---|---|---|---|
| | **Average (%)** | **SEM** | **Average (%)** | **SEM** | **Fold change** | ***p*-Adjusted[a]** |
| H2N3F1 | 0.1 | 0.0 | 0.3 | 0.1 | 3.0 | 0.011 |
| H3N3F1 | 1.3 | 0.2 | 2.0 | 0.1 | 1.5 | 0.017 |
| | **MSI St II** | | **MSI St IV** | | | |
| | **Average (%)** | **SEM** | **Average (%)** | **SEM** | **Fold change** | ***p*-Adjusted[a]** |
| H3N4 | 0.4 | 0.0 | 0.7 | 0.1 | 1.8 | 0.033 |
| H7N6F1 | 0.2 | 0.0 | 0.1 | 0.0 | 0.5 | 0.018 |
| | **MSI St II BRAFwt** | | **MSI St IV BRAFwt** | | | |
| | **Average (%)** | **SEM** | **Average (%)** | **SEM** | **Fold change** | ***p*-Adjusted[a]** |
| H2N3F1 | 0.1 | 0.0 | 0.3 | 0.0 | 3.0 | 0.040 |
| H3N3F1 | 1.3 | 0.2 | 2.2 | 0.2 | 1.7 | 0.016 |
| H4N6F1 | 0.0 | 0.0 | 0.2 | 0.0 | None in St II | 0.018 |
| H7N6F1 | 0.3 | 0.1 | 0.1 | 0.0 | 0.3 | 0.006 |
| | **MSI St II BRAFmut** | | **MSI St IV BRAFmut** | | | |
| | **Average (%)** | **SEM** | **Average (%)** | **SEM** | **Fold change** | ***p*-Adjusted[a]** |
| H2N3F1 | 0.3 | 0.1 | 0.2 | 0.0 | 0.7 | 0.026 |
| | **MSI St IV BRAFwt** | | **MSI St IV BRAFmut** | | | |
| | **Average (%)** | **SEM** | **Average (%)** | **SEM** | **Fold change** | ***p*-Adjusted[a]** |
| H2N3F1 | 0.3 | 0.0 | 0.2 | 0.0 | 0.7 | 0.043 |

[a]Benjamini-Hochberg; F, deoxyhexose; H, hexose; MSI, microsatellite instability; N, N-acetylhexosamine; St, stage; mut, mutated; wt, wild-type

**Supplementary Table S7.** Significantly different acidic monosaccharide compositions between MSI subgroups

| | MSI St II BRAFwt | | MSI St II BRAFmut | | | |
|---|---|---|---|---|---|---|
| | **Average (%)** | **SEM** | **Average (%)** | **SEM** | **Fold change** | ***p*-Adjusted**[a] |
| S1H4N5F1 | 0.3 | 0.1 | 0.0 | 0.0 | None in BRAFmut | 0.032 |
| S2H5N4 | 18.0 | 1.7 | 33.2 | 5.4 | 1.8 | 0.043 |
| S1H5N5F1 | 1.0 | 0.2 | 0.2 | 0.2 | 0.2 | 0.040 |
| S1H6N5 | 0.7 | 0.1 | 0.0 | 0.0 | None in BRAFmut | 0.002 |
| S2H6N5 | 0.4 | 0.2 | 0.0 | 0.0 | None in BRAFmut | 0.045 |
| S3H6N5 | 1.2 | 0.2 | 0.2 | 0.1 | 0.2 | 0.009 |
| S2H6N5F3 | 0.6 | 0.2 | 0.0 | 0.0 | None in BRAFmut | 0.022 |
| | **MSI St II** | | **MSI St IV** | | | |
| | **Average (%)** | **SEM** | **Average (%)** | **SEM** | **Fold change** | ***p*-Adjusted**[a] |
| S1H4N4F1 | 0.3 | 0.1 | 0.8 | 0.1 | 2.7 | 0.010 |
| S2H6N5 | 0.2 | 0.1 | 0.6 | 0.1 | 3.0 | 0.010 |
| S2H7N6F1 | 0.1 | 0.1 | 0.5 | 0.1 | 5.0 | 0.008 |
| | **MSI St II BRAFwt** | | **MSI St IV BRAFwt** | | | |
| | **Average (%)** | **SEM** | **Average (%)** | **SEM** | **Fold change** | ***p*-Adjusted**[a] |
| S1H4N4F1 | 0.4 | 0.1 | 1.0 | 0.2 | 2.5 | 0.040 |
| S1H6N5 | 0.7 | 0.1 | 0.2 | 0.1 | 0.3 | 0.009 |
| | **MSI St II BRAFmut** | | **MSI St IV BRAFmut** | | | |
| | **Average (%)** | **SEM** | **Average (%)** | **SEM** | **Fold change** | ***p*-Adjusted**[a] |
| S1H4N5F1 | 0.0 | 0.0 | 0.5 | 0.2 | None in St II | 0.008 |
| S2H5N4 | 33.2 | 5.4 | 17.5 | 2.3 | 0.5 | 0.045 |
| S1H5N5F1 | 0.2 | 0.2 | 1.3 | 0.1 | 6.5 | 0.016 |
| S1H6N5 | 0.0 | 0.0 | 0.7 | 0.2 | None in St II | 0.003 |
| S2H6N5 | 0.0 | 0.0 | 0.7 | 0.1 | None in St II | 0.001 |
| S1H6N5F2 | 0.0 | 0.0 | 0.3 | 0.1 | None in St II | 0.030 |
| S3H6N5 | 0.2 | 0.1 | 1.2 | 0.3 | 6.0 | 0.020 |
| S2H7N6F1 | 0.0 | 0.0 | 0.5 | 0.2 | None in St II | 0.017 |
| | **MSI St IV BRAFwt** | | **MSI St IV BRAFmut** | | | |
| | **Average (%)** | **SEM** | **Average (%)** | **SEM** | **Fold change** | ***p*-Adjusted**[a] |
| S1H4N4F1 | 1.0 | 0.2 | 0.5 | 0.1 | 0.5 | 0.043 |
| S1H5N5F1 | 0.8 | 0.1 | 1.3 | 0.1 | 1.6 | 0.042 |
| S1H6N5 | 0.2 | 0.1 | 0.7 | 0.2 | 3.5 | 0.018 |

[a]Benjamini-Hochberg; F, deoxyhexose; H, hexose; MSI, microsatellite instability; N, N-acetylhexosamine; St, stage; mut, mutated; wt, wild-type
